# Effect of Pressure, Post-Pressing Time, and Polymerization Cycle on the Degree of Conversion of Thermoactivated Acrylic Resin

**DOI:** 10.1155/2018/5743840

**Published:** 2018-08-15

**Authors:** Rafaella de S. Leão, Sandra L. D. de Moraes, Kátia A. da S. Aquino, Cristina P. Isolan, Bruno G. da S. Casado, Marcos A. J. R. Montes

**Affiliations:** ^1^Department of Restorative Dentistry, Faculty of Dentistry, University of Pernambuco-FOP/UPE, Av. Gal. Newton Cavalcanti 1650, 54753-020 Camaragibe, PE, Brazil; ^2^Department of Prosthodontics, Faculty of Dentistry, University of Pernambuco-FOP/UPE, Av. Gal. Newton Cavalcanti 1650, 54753-020 Camaragibe, PE, Brazil; ^3^Department of Nuclear Engineering, Federal University of Pernambuco (UFPE), Av. Prof. Moraes Rego, 1235-Cidade Universitária, 50670-901 Recife, PE, Brazil; ^4^Department of Restorative Dentistry, Faculty of Dentistry, Federal University of Pelotas (UFPel), R. Gonçalves Chaves, 457-Centro, 96015-560 Pelotas, RS, Brazil

## Abstract

Herein, the effect of different post-pressing times and pressure in two cycles of polymerization on the degree of conversion (DC) of thermally activated acrylic resin (TRRA) is analyzed to optimize the polymerization of this material. After post-pressing for 0, 6, or 12 h, polymerization was performed with or without a pressure of 60 psi (0.41 MPa) in a short (4 h) or a long (11 h) cycle, totaling 12 groups. To determine the DC, PMMA specimens were analyzed by Fourier transform infrared spectroscopy. The influence of each factor alone on the DC was studied by experimental planning. The statistical tests used were three-way ANOVA, *t*-test, Tukey's test, and Levene's test, with a margin of error of 5%. Two groups prepared with post-pressing times of 12 h had the lowest DC (*p* < 0.001). Post-pressing times of 0 and 6 h did not yield statistically different results. Pressure increased the DC in only one group (long cycle +12 h, *p*=0.001). The short cycle resulted in a higher DC than the long cycle in 2 groups (with pressure +0 h, *p*=0.002; without pressure +6 h, *p*=0.015), while the long cycle yielded a statistically higher DC in only one group (with pressure +12 h, *p* < 0.001). The polymerization showed satisfactory DC in all 12 groups. Small differences found among the specimens indicate that the pressure, post-pressing time, and polymerization cycles herein were not influential factors for the DC of PMMA.

## 1. Introduction

Thermoactivated acrylic resins, such as poly (methyl methacrylate) (PMMA), are alloplastic materials used in dentistry and present ease of handling, mechanical resistance, good availability, low thermal and electrical conductivity, low weight, and low cost [1]. PMMA has been mainly used in removable total or removable partial bases since 1930 [2–5]. In addition, they are promising for fabricating internal prostheses and replacing missing bony segments of the skull and face [6–9]. Studies have found that PMMA yields results similar to gold standard materials such as autogenous bone and titanium [1] for these purposes, which makes this material one of the most versatile for the health industry.

Despite its advantages, adverse reactions to PMMA have been reported in the literature [10–12]. The viability of cells exposed to the acrylic resin is influenced mainly by the presence of residual monomers in the samples, that is, by the degree of conversion (DC) achieved in the polymerization reaction [9, 13, 14].

In vinyl polymers, the DC is calculated from the ratio of the concentration of aliphatic C=C double bonds remaining in a polymerized sample to the total number of C=C bonds in the unpolymerized sample, that is, the monomer of origin. Fourier transform infrared spectroscopy (FTIR) is one of the most widely used methods to evaluate the DC [15].

When processing dental prostheses, the most studied parameter affecting DC is the polymerization cycle. Common polymerization methods are microwave energy, autoclave polymerization, and (the most used) water bath [16, 17]. Polymerization can be realized using two cycles: a short cycle and a long cycle [5]. A good acrylic polymerization method is capable of achieving the best physicochemical and biological properties of the acrylic resin, such as hardness, porosity, and monomer release [4].

The influence of other factors such as post-pressing time and the use of pressure during polymerization have not yet been established. Studies on the mechanical properties reported improvements after 12 and 24 h of post-pressing time, supposedly due to an increase of the DC [2, 16]. High pressures (500 MPa) positively influenced the transformation of the monomers into high-molecular-weight polymers by increasing the conversion percentage [18]. However, literary records proving the influence of smaller pressures, which can be applied by typical laboratory-scale equipment, are not yet available.

In view of the lack of information on the correlation of post-pressing time and influence of the pressure on the DC, this study aimed at analyzing the effect of these factors on the conversion of PMMA in two cycles of polymerization. The null hypotheses of this study are as follows: there is no association between post-pressing time and polymerization cycle, and there is no association between presence of pressure and degree of PMMA conversion.

## 2. Materials and Methods

### 2.1. Preparation of Specimens

The specimens were prepared in the same way as in the manufacture of conventional total prostheses. For this, aluminum magazines with the dimensions of 40 × 10 × 2 mm^3^ were manufactured. The base of plastic flasks was partially filled with 150 g of type III gypsum (Herodent, Vigodent Coltene). After gypsum crystallization, the space left between the gypsum and the edge of the base of the muffle was filled with laboratory-grade silicone (Zetalabor, Zhermack), in which the matrices were inserted.

The muffle was closed and a new layer of plaster was poured over it. The resulting mold was filled with a Lucitone 199 acrylic resin at the powder/liquid ratio recommended by the manufacturer (light pink powder: PMMA polymer, pigments, and fiber aesthetics, liquid: methyl methacrylate; Dentsply Indústria e Comércio LTDA, Petrópolis, RJ, Brazil). The muffle was closed and then pressed using a hydraulic press (Reco Hydromatic Press-Reco Dental, Wiesbaden); the applied pressure was slowly increased to 100 kgf·cm^−2^ (9.8 MPa). Subsequently, the specimens were either immediately polymerized (post-pressing time of 0 h) or subjected to post-pressing for 6 h or 12 h prior to polymerization.

The test specimens were polymerized in a pneumatic digital polymerizer (PPD Indústria Digital 08 Fenix; Souza and Marques Pedranópolis, SP, Brazil). The test groups were polymerized with a short or long cycle. For the short cycle, first, water was heated to 70°C (47 min) and this temperature was maintained for 1 h 50 min. Subsequently, the water temperature was raised to 100°C (5 min) and held for 1 h 5 min, leading to a total polymerization time of approximately 4 h. For the long cycle, water was heated to 70°C (40 ± 5 min) and then held at this temperature for 9 h. The water temperature was then raised to 100°C (30 min) and held for 30 min, leading to a total polymerization time of approximately 11 h. To analyze the influence of applied pressure before the polymerization, 60 psi (0.41 MPa) of pressure was applied. Upon completion of the polymerization cycle, the specimens were allowed to cool on the bench; the muffle was then opened and the specimens were removed.

Thus, through the combinations of the studied variables, 12 types of samples were prepared (Table 1). The sample calculation was performed using the program BioEstat 5.0, based on the results of the mean and standard deviation of two groups of 6 specimens each, as detailed in an article by Bural et al. [13].

### 2.2. Analysis of the Degree of Conversion

The degree of conversion of methyl methacrylate (MMA) to PMMA was determined using Fourier transform infrared spectroscopy in the attenuated total reflectance (FTIR-ATR) mode. A Shimadzu Prestige 21 spectrometer (4 cm^−1^ resolution and 16 scans) was used in the absorbance mode. All specimens as well as the nonpolymerized material were analyzed.

To prepare the PMMA samples for the FTIR-ATR analysis, the surface of each polymerized test specimen was sprayed with a tungsten drill; the resulting powder was sieved to obtain a uniform particle size and was placed on the ATR crystal for the test. To prepare the nonpolymerized material, the prepolymerized resin powder was heated at 70°C for 30 h to degrade the benzoyl peroxide and to avoid polymerization reactions of the material after handling with the (liquid) monomer. After mixing the powder (without benzoyl peroxide) and liquid (monomer) following the manufacturer's instructions, the resulting blend was also placed on the ATR crystal.

The DC was determined from the ratio of the absorbance intensity observed for aliphatic C=C at 1635 cm^−1^ and the absorbance intensity of the carbonyl bond C=O (1720 cm^−1^), used as the internal standard of polymerized material for both the polymerized material and the unpolymerized material, according to the following equation [19]:

(1) $$\begin{array}{c} \text{degree of conversion}\left(\%\right)=100\left[1-\frac{\left(A_{1638}/A_{1720}\right)\text{polymerized}}{\left(A_{1638}/A_{1720}\right)\text{unpolymerized}}\right]. \end{array}$$

### 2.3. Statistical Analysis

An experimental design was established to analyze the influence of each factor on the DC. Data were expressed as mean, standard deviation, and coefficient of variation and were inferentially analyzed by three-way ANOVA, one-way ANOVA F test, and Student's *t*-test with equal or unequal variances. In the case of significantly different post-pressing times, Tukey's multiple comparison test was used.

Equality of variances was assessed using the Levene test. The margin of error used in the statistical test decisions was 5%. The data were entered into an EXCEL worksheet, and the statistical calculations were performed with the statistical program SPSS (Statistical Package for the Social Sciences) version 23.

## 3. Results

**Figure 1:**
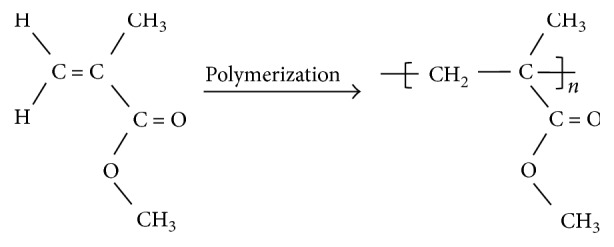
Schematic representation of the conversion reaction of MMA to PMMA. Double bonding of the monomer is broken in the polymerization reaction.

**Figure 2:**
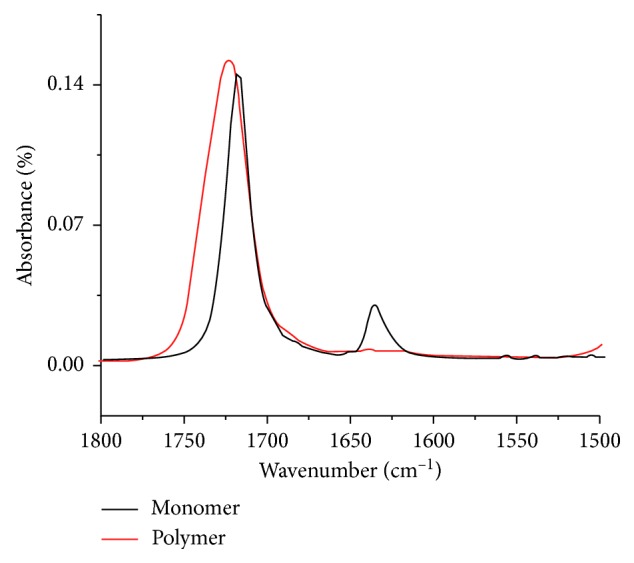
FTIR-ATR spectrum of MMA and PMMA in the region of 1800–1500 cm^−1^. The band at 1635 cm^−1^, referring to the C=C bond, appears in the MMA spectrum and does not appear in the PMMA spectrum, evidencing the conversion.

Figure 1 shows a simple representation of the polymerization of MMA to PMMA.  Figure 2 shows the obtained FTIR-ATR spectra of MMA and PMMA in the 1800–1500 cm^−1^ region. In Table 2, the main FTIR bands attributed to PMMA and MMA according to Silverstein et al. [20] are given.

The DC values (%) and *p* values obtained from the statistical analysis of the DC for each studied variable are presented separately in Tables 3–5. The lowest mean DC (92.66%) was recorded for the long polymerization cycle without applied pressure and with a post-pressing time of 12 h. The mean DC was higher (97.39%) when the post-pressing time was 0 h under otherwise identical conditions.

An ANOVA evaluation of the DC for the 12 groups studied with a factor considering the 12 combinations of the variables is presented in Table 6. By means of Tukey's multiple comparisons test, we verified in which groups the DC mean difference occurred.

## 4. Discussion

This work presents some results that have not yet been reported in the literature. The null hypotheses of this study were accepted: neither the post-pressing time nor the presence or absence of pressure during the polymerization had a statistically significant influence on the DC of MMA to PMMA.

Despite the fact that few studies focus on the influence of pressure on PMMA, the results of this study can be correlated with those found in works by Murakami et al. [18] and Nguyen et al. [21]. It should be noted however that these studies did not use the same pressure conditions. Here, the application of a pressure of 500 MPa resulted in the formation of high-molecular-weight polymers with a high DC. According to Nguyen et al. [21], this result was due to the decrease of intermolecular distance in the material, reducing the free volume and consequently generating a product with a greater density and a reduced number and size of defects. In the present work, we used a smaller pressure of 0.41 MPa due to limitations of our laboratory equipment, and we observed no significant influence of applying this pressure on the DC. Thus, a pressure of 0.41 MPa was not able to increase the molecular weight of PMMA and influence the DC; therefore, it was not possible to verify the statistical difference for the DC values of the majority of the groups, with and without pressure.

Our findings are in agreement with those of Lee et al. [22] who used pressures similar to ours to analyze chemically activated acrylic resins. Note that the same author pointed out that these pressures, while not affecting hardness and DC, could affect porosity. In view of the applications of this material, especially in internal prostheses where the residual monomer levels should be the lowest possible and good mechanical properties are essential, further studies are necessary to determine the minimum pressure required to effectively increase the DC and mechanical properties of the thermally activated acrylic resin. Thus, laboratory-scale equipment capable of applying sufficiently high pressures during the polymerization must be made available.

Regarding the post-pressing time, Consani et al. [23] reported that longer periods promote a lower percentage of residual monomer because of the longer residence time of the resin before being subjected to polymerization. However, our findings differ from these results. A post-pressing time of 12 h (for cycles CC12h, CCP12h, and CL12h) led to statistically lower values of monomeric conversion than shorter post-pressing times. A possible explanation for this could be that any residual monomers would no longer be present in the reaction medium after the 12 h, that is to say, monomers with reaction potential may not have participated in the polymerization due to delay in the start of the reaction. This result directly influences the manufacture of prosthesis, in relation to the logistics of dental laboratories, and has a clinical impact; if the waiting time for polymerization can be decreased, the prosthesis can consequently be installed more quickly.

Longer polymerization cycles were previously found to favor the production of high-molecular-weight polymer chains, improving the hardness and preventing porosity due to the lower amount of residual monomers [16]. However, in our present study, the short cycle led to similar and in some cases even statistically increased DC (Table 2). This may be explained by the fact that the short cycle included a longer period of final boiling at 100°C (1 h) compared with the long cycle (30 min). Longer final boiling is known to be a factor favoring increased conversion of monomers to polymers [17, 24]. Nisar et al. [17] showed that groups that were kept at 100°C for at least 30 min showed less residual monomer content when compared to those that did not undergo this process. These authors obtained the best results for samples that remained at 100°C for longer times, that is, a long heat treatment promoted cross-linking of the polymer chains [5]. Thus, our short cycle that included a longer final boiling period of 1 h was more favorable than our long cycle that included a shorter final boiling period of 30 min. The post-pressing time has an influence on the optimization of the prosthesis surface. A short cycle helps reduce the delivery time of the prosthesis, which is clinically relevant, mainly for hospitalized patients who require installation of internal prostheses, as it reduces the risk of infection.

The polymerization of PMMA is an addition reaction in which, theoretically, no by-products are formed [25, 26]. However, while the DCs of >90% for the twelve groups of specimen analyzed were in accordance with typical results found in the literature [17, 27–29], they are less than 100% and, hence, indicate the presence of residual monomers, as shown in Tables 3–6. This can probably be ascribed to the inhibition of free radicals that are responsible for breaking the carbon-carbon double bonds by reacting with substances other than MMA [22]. The prepolymerized powder used in the preparation of PMMA dentures contains pigments, fibers, and, especially in case of the resin used (Lucitone 199), a higher percentage of plasticizers that improve the impact strength. These additives could react with the free radicals and, thus, be responsible for the presence of residual monomers. The influence of these components is reflected by shifts of the MMA-related FTIR bands when compared to the PMMA-related bands (Figure 2) and is expected because the distinct chemical environments of each molecule promote specific vibrations.

We must note that this work only takes into account the degree of conversion of PMMA. While there is a link between the DC and the biocompatibility as well as the mechanics of this material, the present study should be complemented by cytotoxicity tests as well as measurements of mechanical and physical properties such as impact strength, hardness, surface roughness, and porosity. Only a full evaluation of the material will allow the determination of the best polymerization cycle or the best combinations of variables for the processing of PMMA-based dentures.

## 5. Conclusion

The post-pressing time and application of pressure did not influence the degree of conversion of PMMA. However, these factors may influence other PMMA characteristics such as porosity and mechanical properties, and additional investigative work is required.

## Figures and Tables

**Table 1:** Division of groups according to variables to analyze the degree of conversion.

Polymerization cycle	Post-pressing time		
	Immediate	6 hours	12 hours
Short cycle—no pressure	SC0h	SC6h	SC12h
Short cycle—with pressure	SCP0h	SCP6h	SCP12h
Long cycle—no pressure	CL0h	CL6h	CL12h
Long cycle—with pressure	CLP0h	CLP6h	CLP12h

**Table 2:** Main FTIR bands attributed to PMMA and MMA [20].

Bands (cm^−1^)		Attribution
MMA	PMMA	
2989	2962	Axial deformation of the C-H bond of the -CH_3_ group
2954	2923	Axial deformation of the C-H bond of the -CH_2_ group
1719	1728	Axial deformation of the C=O group of the ester group
1635	—	C=C connection vibration
1438	1434	Symmetrical angular deformation of the group -CH_2_
1380	1381	Angular deformation of the -CH_3_ group
1301	1280	C-O vibration of the trans-conformation ester group
1191	1196	Vibration of the trans-conformation group -O-CH_3_
—	1144	C-C vibration of polymer chain
—	842	C-C of the syndiotactic conformation of the polymer chain
750	751	Asymmetrical angular deformation of the CH_2_ group

**Table 3:** DC as a function of the post-pressing time.

Cycle	Pressure	Post-pressing time			*p* value
		0 h	6 h	12 h	
		Mean ± SD	Mean ± SD	Mean ± SD	
Short	Without	96.30 ± 1.61 (1.67)	97.34 ± 1.19 (1.22)	93.09 ± 4.36 (4.68)	*p* = 0.045^*∗*^
	With	96.55 ± 0.49 (0.51)	95.95 ± 0.98 (1.02)	93.74 ± 1.04 (1.11)	*p* < 0.001^*∗*^

Long	Without	97.39 ± 0.96 (0.99)	95.43 ± 1.07 (1.12)	92.66 ± 1.83 (1.97)	*p* < 0.001^*∗*^
	With	94.68 ± 1.01 (1.07)	95.93 ± 2.20 (2.29)	96.16 ± 0.58 (0.60)	*p* = 0.160

^*∗*^Significant difference; *p* = probability of significance.

**Table 4:** DC as a function of pressure.

Cycle	Pressure	Post-pressing time		
		0 h	6 h	12 h
		Mean ± SD	Mean ± SD	Mean ± SD
Short	Without	96.30 ± 1.61 (1.67)	97.34 ± 1.19 (1.22)	93.09 ± 4.36 (4.68)
	With	96.55 ± 0.49 (0.51)	95.95 ± 0.98 (1.02)	93.74 ± 1.04 (1.11)
*p* value		*p* = 0.736	*p* = 0.052	*p* = 0.729

Long	Without	97.39 ± 0.96 (0.99)	95.43 ± 1.07 (1.12)	92.66 ± 1.83 (1.97)
	With	94.68 ± 1.01 (1.07)	95.93 ± 2.20 (2.29)	96.16 ± 0.58 (0.60)
*p* value		*p* = 0.001^*∗*^	*p* = 0.626	*p* = 0.001^*∗*^

^*∗*^Significant difference; *p* = probability of significance.

**Table 5:** DC for the two different polymerization cycles.

Cycle	Pressure	Post-pressing time		
		0 h	6 h	12 h
		Mean ± SD	Mean ± DP	Mean ± DP
Short	Without	96.30 ± 1.61 (1.67)	97.34 ± 1.19 (1.22)	93.09 ± 4.36 (4.68)
	With	96.55 ± 0.49 (0.51)	95.95 ± 0.98 (1.02)	93.74 ± 1.04 (1.11)

Long	Without	97.39 ± 0.96 (0.99)	95.43 ± 1.07 (1.12)	92.66 ± 1.83 (1.97)
	With	94.68 ± 1.01 (1.07)	95.93 ± 2.20 (2.29)	96.16 ± 0.58 (0.60)

*p* value: without polymerization		*p* = 0.187	*p* = 0.015^*∗*^	*p* = 0.830

*p* value: with polymerization		*p* = 0.002^*∗*^	*p* = 0.985	*p* < 0.001^*∗*^

^*∗*^Significant difference; *p* = probability of significance.

**Table 6:** DC for each group.

Cycle	Pressure	Post-pressing time		
		0 h	6 h	12 h
		Mean ± DP (CV%)	Mean ± DP (CV%)	Mean ± DP (CV%)
Short	Without	96.30 ± 1.61 (1.67) (AB)	97.34 ± 1.19 (1.22) (AC)	93.09 ± 4.36 (4.68) (AB)
	With	96.55 ± 0.49 (0.51) (ABC)	95.95 ± 0.98 (1.02) (AB)	93.74 ± 1.04 (1.11) (BE)

Long	Without	97.39 ± 0.96 (0.99) (AC)	95.43 ± 1.07 (1.12) (AB)	92.66 ± 1.83 (1.97) (BD)
	With	94.68 ± 1.01 (1.07) (AE)	95.93 ± 2.20 (2.29) (AB)	96.16 ± 0.58 (0.60) (AB)

Letters represent statistical similarity (obs: consider only one letter, not necessarily the set of letters).

## Data Availability

The data referring to the degree of conversion values of each sample used to support the findings of this study are available from the corresponding author upon request.
